# Core Biopsy Diagnosis of ALK Positive Inflammatory Myofibroblastic Tumor of Lung: An Interesting Case

**DOI:** 10.5146/tjpath.2018.01446

**Published:** 2020-05-15

**Authors:** Ritesh Sachdev, Ishani Mohapatra, Shalini Goel, Kulbir Ahlawat, Neelam Sharma

**Affiliations:** Department of Pathology, Lab Medicine and Transfusion Medicine, Medanta - The Medicity Hospital, Sector 38, Gurgaon, India; Department of Radiology, Medanta - The Medicity Hospital, Sector 38, Gurgaon, India; Department of Medical Oncology, Medanta - The Medicity Hospital, Sector 38, Gurgaon, India

**Keywords:** Anaplastic lymphoma kinase, Inflammatory myofibroblastic tumor, Lung, Immunohistochemistry, Core needle biopsy

## Abstract

Inflammatory myofibroblastic tumor (IMT) of lung is a rare tumor, accounting for ~0.7% of all lung tumors with varied clinical and radiological presentations. The origin of this tumor is unknown but some studies suggest that it might be a true neoplasm as some mutations on chromosome 2p23 of anaplastic lymphoma kinase (ALK) have been found to be related to this tumor. The morphology of IMT is quite vague and the histopathological diagnosis is predominantly given on excision specimens; in fact, only 6.3% of cases are diagnosed based on analysis of biopsy specimens alone. We illustrate a case of IMT diagnosed in a young male on core biopsy, where the case presented with a large tumor in the lung with metastases to multiple sites that was hence unresectable. Post 3 months of treatment with Crizotinib, there was significant reduction in the tumor size. Another interesting finding was that the ALK immunostain, which helped immensely in the diagnosis, was appreciated better on the Ventana platform rather than on the Dako platform.

## INTRODUCTION

Inflammatory myofibroblastic tumor (IMT) of lung is a rare tumor accounting for ~0.7% of all lung tumors. It has a varied clinical and radiological presentation. Clinically, the patients usually present with respiratory symptoms such as cough, dyspnoea, fever, fatigue and haemoptysis. Rarely, features of airway obstruction are seen. Radiologically, this tumor can be cystic or homogeneous, endobronchial or parenchymal, and with or without clear margins. The origin of this tumor is unknown but some studies call it a reactive process while a few of them suggest that it might be a true neoplasm as some mutations on chromosome 2p23 of anaplastic lymphoma kinase (ALK) have been found to be related to this tumor. The morphology of IMT is quite vague and the histopathological diagnosis is predominantly given on excision specimens; in fact, only 6.3% of cases are diagnosed based on analysis of biopsy specimen alone (1–4). We hereby present a case of IMT in a young male with features of airway obstruction, where the case had multiple tumors involving the lung, mediastinum, liver and bone, and was diagnosed on core biopsy from the lung at our hospital.

## CASE REPORT

**Figure 1:**
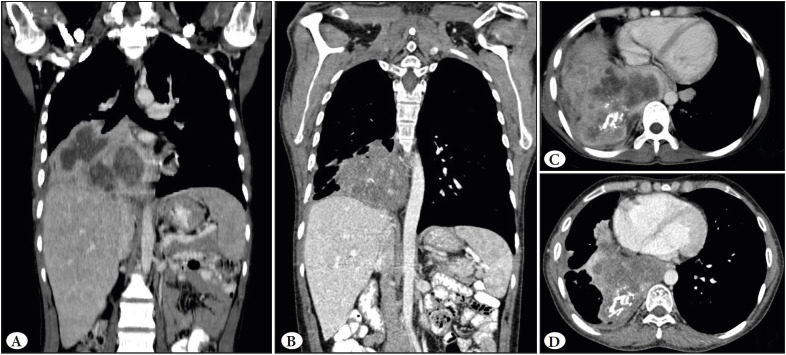
CECT chest and abdomen pre-treatment (A and C) and 3 months post treatment (B and D). **A)** Coronal view of the large heterogeneously enhancing lesion which involved the right middle and lower lobe of the lung, impinging on the supero-posterior surface of the liver. **C)** Axial view of the large mass involving the right middle and lower lobe of the lung with coarse calcification, involving the mediastinal pleura and pericardium and extending into the mediastinum. **B&D)** Coronal and axial view showing reduction in the size of the tumor.

**Figure 2:**
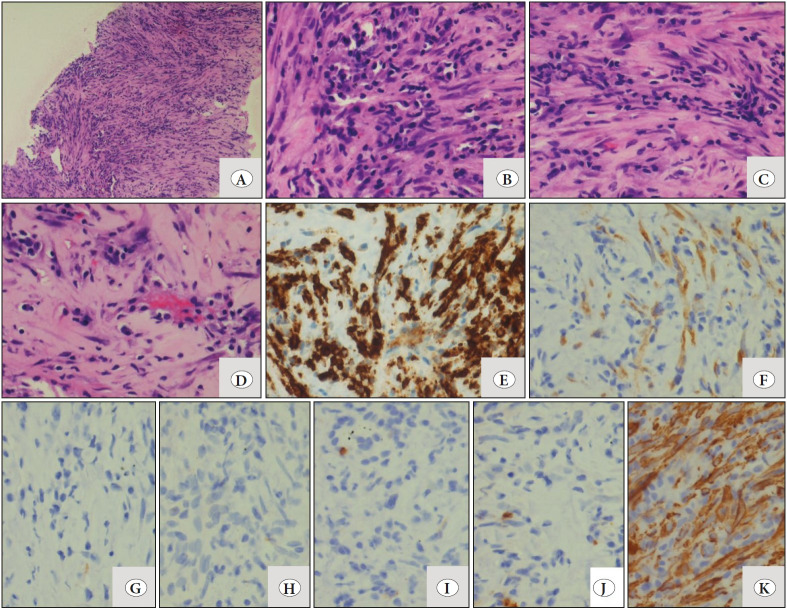
**A)** Low magnification shows core biopsy with proliferation of bland spindle cells (H&E; x20). **B-D)** High magnification shows different areas observed in the biopsy such as areas with predominance of lymphoplasmacytic infiltrate and spindle cells arranged in long fascicles. (H&E; x40). **E-F)** IHC shows comparison of ALK immunostains, on both the Dako platform using monoclonal mouse anti-human CD246 antibody (clone ALK1) which showed focal positivity with weak intensity (F; x40) and the Ventana platform using monoclonal rabbit anti-human CD246 antibody (clone D5F3) reported as strong positive (E; x40). Spindle cells are negative for the following: **G) **Desmin **H)** CK **I)** CD117 and **J)** S-100 (IHC; x40). **K)** SMA positivity in neoplastic cells (IHC; x40).

A 31-year-old male came with complaints of dry cough, breathlessness, weight loss and loss of appetite for the last 3 months. CECT (Contrast enhanced computed tomography) chest was done at an outside facility and the differentials considered were adenosquamous carcinoma, mesothelioma or solitary fibrous tumor. Core biopsy was also done, and a possibility of adenosquamous carcinoma was favored in view of the clinical and radiological findings. Due to paucity of tissue, immunohistochemistry (IHC) was not attempted. The patient came to our institute for a second opinion and treatment. On examination, the patient had pallor and a performance status of 2. CECT chest and abdomen were repeated and revealed a large heterogeneously enhancing lesion which involved the right middle and lower lobe and measuring approximately 12.9 (AP) x 11.9 (TR) x 9.6 (CC) cm with presence of coarse calcification (Figure 1). The lesion was closely abutting the right upper lobe bronchus with involvement of the mediastinal pleura and pericardium, and extending into the mediastinum. The fat plane between the lesion and the liver were also effaced at places with likely diaphragmatic and liver involvement. The mass was impinging on the supero-posterior surface of the liver (Figure 1). The lesion was seen closely abutting the descending aorta, right atrium, inferior vena-cava, right main pulmonary artery, right sided pulmonary veins, left inferior pulmonary vein and left atrium (Figure 1). There were multiple heterogeneously enhancing soft tissue pleural and parenchymal lesions seen in both lung fields, with the largest measuring approximately 4.2x2.3x1.9 cm. There were multiple heterogeneously enhancing lymph nodes seen in the pre-tracheal, para-tracheal and right hilar regions, with the largest measuring 1.8x1.5 cm. The liver measured 21.7 cm in its cranio-caudal extent with ill-defined hypodense areas and peripheral enhancement in segment VI. The CECT findings were suspicious for malignant etiology with lung, liver, bony and mediastinal nodal metastasis. Other investigations showed normal renal and liver function tests, normal thyroid function tests, non-reactive viral markers and normal coagulation studies. On the basis of the clinical and radiological inputs, a diagnosis of metastatic carcinoma involving the liver and lymph nodes with primary from the lung was considered. Complete blood count revealed anemia (Hemoglobin: 5.2g/dL), mild leukocytosis (14.02x103/cumm) and mild thrombocytosis (717x103/cumm). Peripheral smear showed no abnormal cells. Bone marrow examination was also performed and showed a fair number of plasma cells with a polyclonal pattern of distribution. In view of the above findings, serum protein electrophoresis was also done and no “M” band was seen. Under CT guidance, a core biopsy of right upper lobe lung nodule was done under local anaesthesia using an 18G biopsy needle (multiple cores measuring 1.0-1.7 cm). These core biopsies showed proliferation of bland spindle cells (Figure 2), at places arranged in long fascicles, in an inflammatory background consisting of many plasma cells with scattered lymphocytes (Figure 2). Native lung parenchyma was seen within the lesion. The closest differential considered was IgG4 related disease due to the bland nature of the spindle cells in the background of plasma cells. Other differentials such as leiomyosarcoma and mesothelioma were also considered but were ruled out due to the bland morphology and presence of inflammatory background. On IHC, these spindle cells expressed ALK (Figure 2) and SMA (Figure 2), whilst they were negative for desmin, cytokeratin, CD117, S-100 (Figure 2), CD21 and IgG4 immunostain. ALK immunostaining was done on the Dako platform using monoclonal mouse anti-human CD246 antibody (clone ALK1) which showed focal positivity with weak intensity (Figure 2). ALK immunostain was then also performed on the Ventana platform using monoclonal rabbit anti-human CD246 antibody (clone D5F3), which showed a higher proportion of cellular staining with higher intensity in the spindle cells (Figure 2). A diagnosis of an inflammatory myofibroblastic tumor (IMT) of the lung was given. ALK gene re-arrangement by FISH (Florescent in-situ hybridization) was attempted but yielded a non-contributory result due to the exhaustion of the tissue block after performing the immunohistochemistry.

Following diagnosis, the patient was started on Crizotinib and has till date completed 3 months of therapy. CECT findings on comparison with the previous scan reveal interval reduction in the previously noted right lung mass and multiple lung nodules, now measuring approximately 10.9 (AP) x 9.5 (TR) x 8.6 (CC) cm (previously measured 12.9 (AP) x 11.9 (TR) x 9.6 (CC) cm) (Figure 1).

## DISCUSSION

Inflammatory Myofibroblastic Tumor (IMT), previously called inflammatory pseudotumor, plasma cell granuloma, histiocytoma or fibroxanthoma, was first described in the lung in 1939. The lung being the primary site, it has also been described in other extra pulmonary sites such as the spleen, lymph nodes, esophagus, stomach, salivary glands, breast, epididymis, central nervous system, and soft tissues (1). Matsubara et al. used the term inflammatory pseudotumor and described three subgroups depending on the cell type encountered in a mass: organizing pneumonia, fibrous histiocytoma, and lymphoplasmocytic type. In all three types, an intra-alveolar organizing inflammation was observed and they hypothesized that an inflammatory pseudotumor probably originates from an organizing pneumonia. (2). In 1990, the term IMT was used based on the morphology and the immunohistochemical features of the spindle cells in these lesions resembling those of myofibroblasts. Most reports suggested an inflammatory or infective pathogenesis (~30% of cases are associated with respiratory infections) with an exaggerated response to tissue damage and subsequently pseudotumor formation. There were reports that suggested a true neoplastic nature with clonal chromosomal changes in chromosome 2. These changes are found in region 2p23 with activation of the ALK gene proving that at least a few IMT’s were indeed true neoplasms rather than reactive processes (1,3,4). IMT shows a slight predominance in children and young adults, as seen in our case (1). Usually the patients present with nonspecific symptoms such as cough, dyspnea, hemoptysis, chest pain, fever and fatigue. Weight loss and anorexia are rare (1). However, the present case had both weight loss and loss of appetite along with respiratory symptoms and airway obstruction, suggesting the spread of the disease. Radiologically, the tumor has varied presentations ranging from a single nodule to multiple nodules (rare) either limited to the lung or rarely involving the pleura, diaphragm, mediastinum or liver. On CT scan, the tumor had a heterogeneous appearance with solid and cystic areas, small calcifications and irregular borders, and thus a wide range of differentials including metastatic tumors were considered (1,4). Lymphadenopathy is an uncommon finding but extensive lymphadenopathy was noted in our case (4). Radiology, fine needle aspiration, or tru-cut biopsies are often not able to distinguish between an IMT, a sarcoma, or other low-grade malignancies (4). According to various studies, the diagnosis has proven to be very difficult and almost only possible after complete resection of the tumor, with only ~6.3% cases reported being diagnosed on biopsy alone (3,4). The morphology of the biopsy was largely non-specific with presence of spindle shaped myofibroblasts along with inflammatory cells including plasma cells. The closest differential considered was IgG4 related disease in view of the presence of plasma cells. However, the IgG4 immunostain was negative, thus excluding this lesion in the present case. The myofibroblasts were positive for SMA and ALK (both on the Dako and Ventana platform). Coffin et al. studied 59 cases of IMT and noted that cytoplasmic ALK reactivity was seen in cases with clonal changes in chromosome 2, as observed in 56% of cases (5). ALK immunostaining was performed on both platforms, Dako and Ventana, with different antibody clones. The rabbit monoclonal anti-human CD246 antibody (clone D5F3) used on the Ventana platform was found to be staining a higher proportion of cells (>50%) with higher intensity (Grade 3) than the mouse monoclonal anti-human CD246 antibody (clone ALK1) on the Dako platform. Taheri et al. in their study showed that D5F3 antibody stained 76% and ALK1 antibody stained 72% of IMTs. Compared to staining with ALK1, D5F3 stained a higher proportion of cases extensively (>50% cells) (76% vs. 28%) and with high intensity (grade 3; 76% vs 0%) (6). This was noted in the present case. The Ventana platform was new in our institute and this was the first case where the ALK immunostain was repeated on the Ventana as well, thereby confirming the diagnosis on the limited quantity of the tissue sample received.

ALK-negative IMTs occurred in older patients and had greater nuclear pleomorphism, atypia, and atypical mitoses, and also metastasized widely. ALK reactivity was associated with local recurrence, but not distant metastasis, which was confined to ALK-negative lesions (5). However, in our case all the features were seen along with involvement of the adjacent sites (liver, bone and lymph nodes). Complete resection of the tumor is considered the treatment of choice with the prognosis depending on the tumor size (less than or equal to 3 cm) and ALK reactivity (3,5). In our case, the mass was large and hence chemotherapy was started as the treatment modality. Coffin et al. have concluded that ALK reactivity may be a favorable prognostic indicator in IMT (5). Reduction in the size of the tumor (~2 cm) has been noted post 3 months of therapy, which points towards the positive ALK reactivity seen in this tumor. The overall 3-year survival rate is about 82% and the overall 5-year survival rate is about 74% (3,4).

This case posed a diagnostic challenge as the radiology and clinical picture was suggestive of malignancy and on histopathology only core biopsy was received on which the architecture of the lesion could not be appreciated. IMT is predominantly a morphological diagnosis based on the architecture of the spindle cells and is usually difficult to diagnose on core biopsies; a strong vigilance and in-depth knowledge of this entity along with a wide variety of immunohistochemical markers therefore helps in reaching a conclusive diagnosis. The novel anti-ALK rabbit monoclonal antibody (D5F3 clone) demonstrates a much higher proportion of cell staining with a higher intensity, thus giving a superior overall performance of ALK protein in IMT. 

## Conflict of Interest

The authors declare no conflict of interest
